# Comprehensive Improvement of Various Features of Cu–Cd Ferrites (Cu_0.5_Cd_0.5_Fe_2–*x*_Ce_*x*_O_4_) by Cerium (Ce^3+^) Ion Substitution

**DOI:** 10.1021/acsomega.3c03993

**Published:** 2023-10-25

**Authors:** Muneeba Fatima, Muhammad Sajjad Ul Hasan, Maria Akhtar, Nicola Morley, Nasir Amin, Atta ur Rehman, Muhammad Imran Arshad, Mongi Amami, Bisma Yaqub, Safa Ezzine

**Affiliations:** † Department of Physics, Government College University, Faisalabad 38000, Pakistan; ‡ Department of Physics, The University of Lahore, Lahore 54000, Pakistan; § Department of Materials Science and Engineering, The University of Sheffield, Sheffield S1 3JD, U.K.; ∥ Biophysics Group, Department of Physics and Astronomy, University College London, Gower Street, London WC1E 6BT, U.K.; ⊥ UCL Healthcare Biomagnetics and Nanomaterials Laboratories, 21 Albemarle Street, London W1S 4BS, U.K.; # Department of Chemistry College of Sciences, King Khalid University, P.O. Box 9004, Abha 61412, Saudi Arabia; ∇ Department of Physics, Government College Women University, Faisalabad 38000, Pakistan

## Abstract

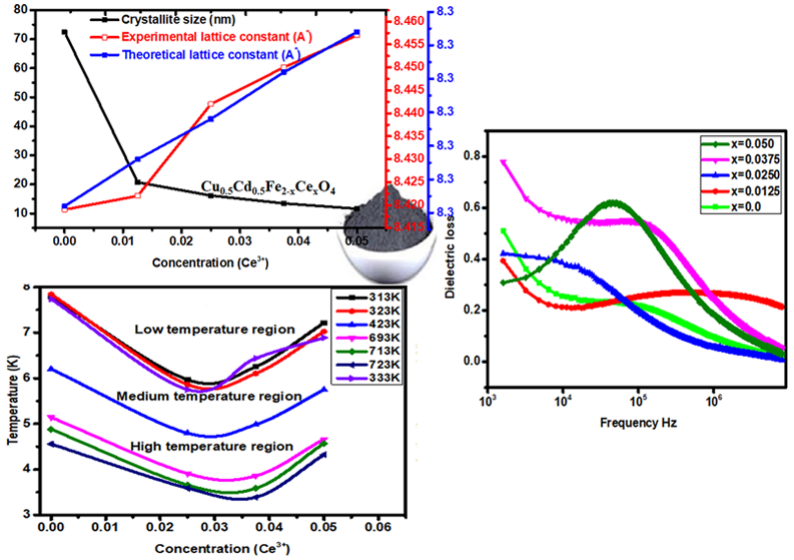

Cerium (Ce^3+^) substitution in Cu–Cd spinel nanoferrites with the compositional formula Cu_0.5_Cd_0.5_Fe_2–*x*_Ce_*x*_O_4_ (*x* = 0.0, 0.0125, 0.0250, 0.0375, 0.050) was performed by the hydrothermal route. The structural, morphological, optical, electrical, and dielectric properties of Ce-substituted Cu–Cd ferrites were explored. X-ray diffraction revealed the single-phase cubic structure of all nanoferrites. The average crystallite size (72.42–11.61 nm) and lattice constant (8.419–8.449 Å) were observed for the synthesized ferrites. The surface shapes of particles were determined by scanning electron microscopy. The substitution was also verified by Fourier transform infrared spectroscopy and ultraviolet–visible spectrophotometry. The semiconducting behavior of ferrites was determined from their electrical properties, such as direct current (DC) electrical resistivity. The Curie temperature was observed at 523 K temperature for all nanoferrites. The dielectric constant and dielectric loss significantly indicated the reducing behavior with an increase in the cerium concentration. The sample Cu_0.5_Cd_0.5_Fe_1.975_Ce_0.025_O_4_ resulted in the lowest optical bandgap energy, DC resistivity, and dielectric losses. The nature of the electrical resistivity and dielectric constants indicate that the designed materials are highly appropriate for the design of microwave gadgets.

## 1 Introduction

Nanomaterials have become the most popular progressing field of research in the past couple of decades. Various researchers have worked on the development of novel nanometal oxides due to their special-sized support properties.^1,2^ Soft ferrites with the chemical formula AB_2_O_4_ (A denotes a divalent cation and B represents iron) have shown immense technical significance due to their magnetic and electrical characteristics.^3^ Nanoferrites exhibit high effectiveness, economy, and appropriate dielectric losses and therefore have prospective functions in memory core and microwave gadgets.^4^ In addition, nanoferrites have various uses in electrical and electronic fields, including magnetic resonance imaging (MRI), microwave absorbance, magnetic fluids, radio-frequency (RF) circuits, power transformers, electromagnetic interference, antennae, radars, and pesticides.^5−8^ The fabrication technique, chemical compounds, particle size, doping, concentration, divalent and trivalent iron ions, and their sharing among tetrahedral and octahedral lattice sites influence the electrical and magnetic characteristics.^9^ The insertion of diverse cations into nanoferrites induces changes in electrical properties. The inclusion of rare earth metals in spinel ferrites to improve electromagnetic properties has been described by several researchers. Dixit et al. prepared cerium-doped nickel nanoferrites by the coprecipitation scheme and determined an irregular magnetic moment with an increasing concentration of cerium.^10^ Mustafa et al. synthesized Ce-inserted CoCr_0.04_Ce_*x*_Fe_1.96–*x*_O_4_ (0 < *x* < 0.1) soft ferrites by the coprecipitation process and discovered reductions in the saturation magnetization with increasing Ce content. They suggested that the fabricated materials were excellent candidates for electromagnetic technology.^11^ Pawae et al. designed Ce-added CuCe_*x*_Fe_2–*x*_O_4_ (*x* = 0.0, 0.04, 0.06, 0.08) ferrites by a sol–gel auto-combustion process and determined their semiconducting nature.^12^ Akhtar et al. fabricated Ce-substituted Gd nanoferrites by applying a sol–gel scheme and found a reduction in saturation magnetization with an increase in Ce concentration.^13^ The partial substitution of Fe by Ce seems to be appropriate to expand the electromagnetic properties. Suzauddulah et al. synthesized Ni-substituted soft ferrites and found an increase in conductivity with an increase in frequency. They also observed that the nanoferrites are suitable candidates for electronic devices.^14^ Er et al. designed Co-inserted Ag–Pd nanotubes and suggested that the nanotubes are applicable in direct formic acid fuel cells.^15^ Abdullah-Al-Mamun et al. prepared Er-substituted Co ferrites by the application of a sol–gel process and calculated a saturation magnetization of 95.92 emu/g for pure cobalt ferrites.^16^ Hossain et al. also synthesized cobalt ferrites and determined the remanence to be less than 0.5. They concluded that the ferrites were suitable for magnetic devices.^17^

In this study, we investigated the influence of the rare earth element Ce^3+^ on Cu–Cd ferrites, focusing on their structural, optical, electrical, and magnetic properties. The optical bandgap energy was determined to range from 3.22 to 2.68 eV. This finding contrasts with Hussain et al.’s research, where Ce^3+^-doped ferrites showed a bandgap range of 4.20–2.18 eV.^18^ Moreover, our results demonstrated a decrease in the direct current (DC) electrical resistivity with increasing temperature, whereas Malana et al. reported a decrease in resistivity as a function of temperature in their work.^9^ Furthermore, we observed variable behavior for the dielectric loss, differing from Ishaque et al.’s findings, which revealed a decrease in the dielectric constant and loss with the enhancement of yttrium ferrites.^19^ In our research, the dielectric loss exhibited varying trends, indicating unique characteristics in the Ce^3+^-doped Cu–Cd ferrites. Furthermore, Cu–Cd nanomaterials showed decreasing dielectric constant and dielectric losses, as reported by Hashim et al.^20^ Soft ferrite nanomaterials play vital roles as antibacterials. The content of nickel introduced may also affect the Cu–Cd ferrites.^21^ The Cu–Cd ferrites with an inverse spinel structure contain the rare earth element (Ce^3+^) incorporated into octahedral sites. M. A. Almessiere et al. observed a reduction in saturation magnetization as the concentration of Ce^3+^ increased in Mn–Zn ferrites. They proposed that such materials could exhibit anticancer and antibacterial activities.^22^ Hashim et al. also confirmed a decline in saturation magnetization with increasing Ce^3+^ content.^23^ Moreover, Shirsath et al. reported that the addition of cerium led to an increase in DC resistivity, observing the maximum resistivity of 5.20 × 10^6^ Ohm·cm.^24^ However, in our research, we observed a resistivity of 5 × 10^7^ Ohm·cm, which is higher than those in previous findings. In addition, various other researchers reported a decrease in saturation magnetization with increasing Ce^3+^ content.^25^

In the current study, Cu_0.5_Cd_0.5_Fe_2–*x*_Ce_*x*_O_4_ (Ce-doped Cu–Cd ferrites) (where *x* = 0.0, 0.0125, 0.0250, 0.0375, 0.050) nanorods were synthesized by the hydrothermal method. The hydrothermal synthesis technique is adopted because of its simplicity, economy, and absence of impurities.^26,27^ To the best of our knowledge, the consequences of Ce substitution on the structural, optical, and electrical characteristics of Cu–Cd spinel ferrites have not been formally discovered. Furthermore, this study endeavors to correlate the structural variations of the fabricated nanomaterials with Ce concentration based on X-ray diffraction (XRD), UV–vis spectroscopy, Fourier transform infrared (FTIR) spectroscopy, and *I*–*V* and LCR performances.

## 2 Experimentation

The chemicals utilized for the production of Ce-doped Cu–Cd nanoferrites included copper nitrate (Cu(NO_3_)_2_·3H_2_O), cadmium nitrate (Cd(NO_3_)_2_·4H_2_O), iron nitrate (Fe(NO_3_)_3_·9H_2_O), and cerium nitrate Ce(NO_3_)_3_·6H_2_O. Stoichiometric ratios (Table 1) of these chemicals were dissolved in ethanol separately; after stirring for half an hour at 0 °C and 400 rpm, the solutions of all of the chemicals were transferred into a single beaker. The mixtures were placed in a Teflon-lined stainless steel autoclave for 24 h at 180 °C and cooled down to ambient temperature. Precipitates were collected using filter paper. For drying purposes, the specimens were set in an electric oven for 48 h at 80 °C and then ground for 1 hour. All specimens were positioned in the furnace at 750 °C for 8 h, followed by grinding again. After this, the materials were characterized using different techniques. A hydraulic press machine was used to create the pellets at 13,000 psi pressure.

**Table 1:** Number of Moles of All Reagents Used in the Synthesis of Ce-Doped Cu–Cd Ferrites

*X*	composition	Cu_0.5_	Cd_0.5_	Fe_2–*x*_	Ce*_x_*
0.0	Cu_0.5_Cd_0.5_Fe_2_O_4_	1.208	1.5425	8.08	0.0
0.0125	Cu_0.5_Cd_0.5_Fe_1.9875_Ce_0.0125_O_4_	1.208	1.5425	8.0295	0.054
0.250	Cu_0.5_Cd_0.5_Fe_1.975_Ce_0.025_O_4_	1.208	1.5425	7.07	1.085
0.375	Cu_0.5_Cd_0.5_Fe_1.9625_Ce_0.0375_O_4_	1.208	1.5425	6.565	1.628
0.050	Cu_0.5_Cd_0.5_Fe_1.95_Ce_0.05_O_4_	1.208	1.5425	7.878	0.217

### 2.1 Characterizations

The X-ray diffraction (Bruker D8) method with a Cu Kα source with λ = 1.5406 Å was used to verify the structure of ferrites. A UV–vis spectrophotometer model V-730 with 190–1100 nm was applied to investigate the optical bandgap energy. FTIR spectroscopy (Perkin) was performed to determine the absorption bands of tetrahedral and octahedral sites. The two-probe *I*–*V* apparatus, including Keithley software, was employed to establish the DC resistivity of soft ferrites. An LCR meter was used to study the AC conductivity and dielectric loss.

**Figure 1:**
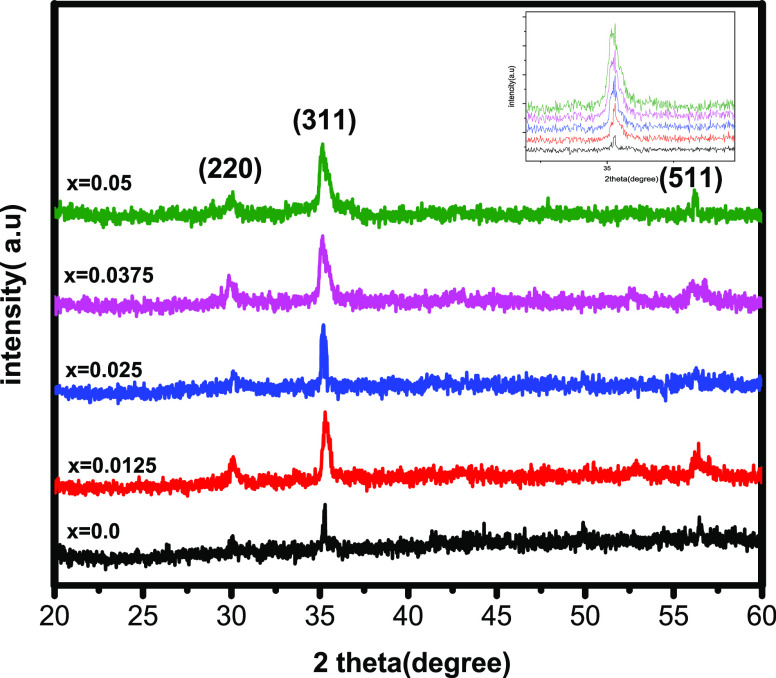
XRD patterns of Cu_0.5_Cd_0.5_Fe_2–*x*_Ce_*x*_O_4_ (*x* = 0.0, 0.0125, 0.025, 0.0375, 0.05) nanoferrites.

## 3 Results and Discussion

### 3.1 Structural Analysis

**Figure 2:**
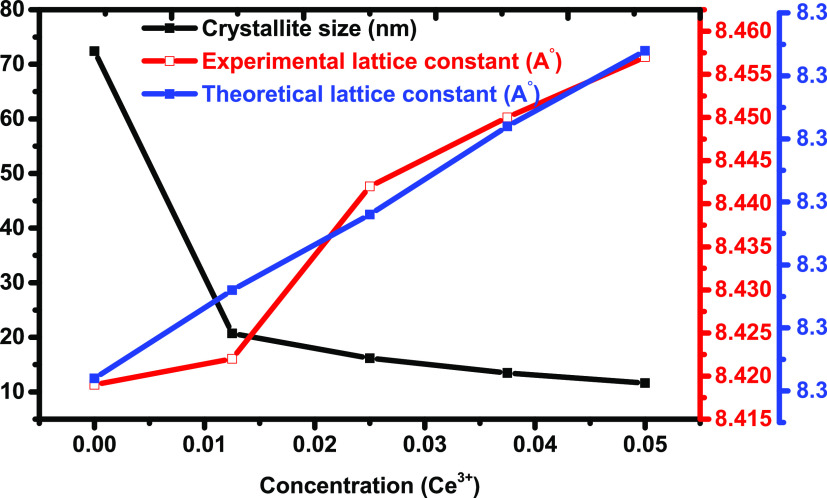
Graph of concentration (Ce^3+^) vs crystallite size, experimental lattice constant (*a*_exp_), and theoretical lattice constant (*a*_th_) for Ce-doped Cu–Cd ferrites

XRD spectra of Cu_0.5_Cd_0.5_Ce_*x*_Fe_2–*x*_O_4_ (*x* = 0.0, 0.0125, 0.025, 0.0375, and 0.05) nanoferrites are presented in Figure 1. From the (220), (311), and (511) peaks and PDF 72-1720, it was verified that single-phase spinel ferrites were produced. The sharpest peak in all XRD patterns is (311). Scherrer’s equation  was applied to calculate the average nanoferrite crystallite size,^28^ where *k* = 0.9, λ = 1.542 Å, β is the fwhm of the observed peaks, and θ is the Bragg angle. The average crystallite size was observed in the range of 72.42–11.61 nm for *x* = 0.0–0.05 nanoferrites, correspondingly, as shown in Table 2. Thus, the outcomes indicate that the particles are in the nanoscale range and have a cubic arrangement. A rapid decrease in the crystallite size is observed from 72.42 nm (*x* = 0.0) to 20.72 nm (*x* = 0.0125) nanoferrites. During the formation of soft ferrites, nucleation and subsequent grain growth occur. If the nucleation rate is favored over the grain growth rate, then smaller crystallites will be formed, resulting in a reduction in size. Also, the introduction of additives such as Ce^3+^ influenced the crystallization behavior of soft ferrites. The dopant may act as a nucleation site or hinder grain growth, leading to the formation of smaller crystallites. Such factors may cause a rapid decrease in the crystallite size. The lattice parameters^29^ were observed using the relationship , and the lattice parameters were enhanced from 8.419 to 8.457 Å with the introduction of cerium; the trend inferred in the case of ionic radii is Fe^3+^ (0.645 Å) and Ce^3+^ (1.034 Å). When iron is replaced by cerium, the lattice constant increases. Furthermore, the sharpest peak (311) shifts toward a lower angle, as shown in the inset of Figure 1. The behaviors exhibited by the crystallite size and lattice constants are depicted in Figure 2. The average volume of a unit cell, X-ray, and bulk densities (*d*_*x*_ and *d*_b_)^30^ are given in Table 2. The cell volume showed a trend similar to that of the lattice constant. The X-ray density of the fabricated nanoferrites decreased as the concentration of cerium increased. This is due to the lower molar mass of cerium than that of iron. The difference in densities may be due to the addition of some impurities during the synthesis process. “*d*_b_” is reduced due to dissimilarities in the atomic weights of Ce^3+^ and Fe^3+^, resulting in variations in porosity.^18,31^ The reducing behaviors of both densities (*d*_*x*_ and *d*_b_) and porosity are shown in Table 2. Various other parameters, including the polaron radius and hopping lengths for tetrahedral and octahedral sites, were resolved.^32^ The polaron radius exhibited an increasing trend with an increase in Ce content, as shown in Table 2. The increase in the values of the polaron radius indicates that a larger potential is required to transfer the charge carriers among the cationic sites. Both *L*_A_ and *L*_B_ showed increasing trends. This means that a larger energy is required for the charge carriers to travel between the cationic sites, as confirmed by the results of the polaron radius.^33^

**Table 2:** Various Calculated XRD Parameters for Cu_0.5_Cd_0.5_Fe_2–*x*_Ce_*x*_O_4_ (*x* = 0.0, 0.0125, 0.025, 0.0375, 0.05) Nanoferrites

parameters	*x* = 0.0	*x* = 0.0125	*x* = 0.0250	*x* = 0.0375	*x* = 0.05
crystallite size (nm)	72.42 (+0.1, −0.1)	20.72 (+0.3, −0.3)	16.16 (+0.02, −0.02)	13.47 (+0.2, −0.2)	11.61 (+0.1, −0.1)
lattice constant (Å)	8.419 (±0.1125)	8.422 (±0.1090)	8.442 (±0.1270)	8.450 (±0.1240)	8.457 (±0.1245)
unit cell volume (Å^3^)	596.73	597.37	601.63	603.35	604.85
X-ray density (g/cm^3^)	5.982	5.701	5.653	5.634	5.634
bulk density (g/cm^3^)	4.455	4.051	4.044	4.037	4.031
porosity (%)	25.52	28.93	28.45	28.33	28.45
polaron radius (Å)	0.5421	0.5427	0.5466	0.5481	0.5495
hopping length *L*_A_ (Å)	3.1571	3.1582	3.1657	3.1687	3.1713
hopping length *L*_A_ (Å)	2.1047	2.1055	2.1105	2.1125	2.1142

#### 3.1.1 Mechanical Parameters

Various mechanical parameters were determined, including the surface area, packing factor, strain, and dislocation density, from the XRD data. The following relations were used to investigate the parameters^34^
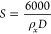
1
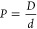
2
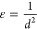
3
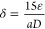
4where ρ_*x*_, *D*, and *d* denote the X-ray density, crystallite size, and interplanar spacing, respectively. Table 3 reveals the results of all of the mechanical factors.

**Table 3:** Different Calculated Mechanical Parameters for Cu_0.5_Cd_0.5_Fe_2–*x*_Ce_*x*_O_4_ (*x* = 0.0, 0.0125, 0.025, 0.0375, 0.05) Nanoferrites

compositions	specific surface area (m^2^/g)	packing factor (p)	strain (ε)	dislocation density (g/m^3^)
Cu_0.5_Cd_0.5_Fe_2_O_4_	15.85	27.52	0.1444	0.0035
Cu_0.5_Cd_0.5_Fe_1.9875_Ce_0.0125_O_4_	56.37	7.87	0.1445	0.0124
Cu_0.5_Cd_0.5_Fe_1.975_Ce_0.025_O_4_	73.36	6.15	0.1449	0.0159
Cu_0.5_Cd_0.5_Fe_1.9625_Ce_0.0375_O_4_	90.01	5.13	0.1452	0.0191
Cu_0.5_Cd_0.5_Fe_1.95_Ce_0.05_O_4_	106.83	4.42	0.1453	0.0222

#### 3.1.2 Cation Distribution

**Figure 3:**
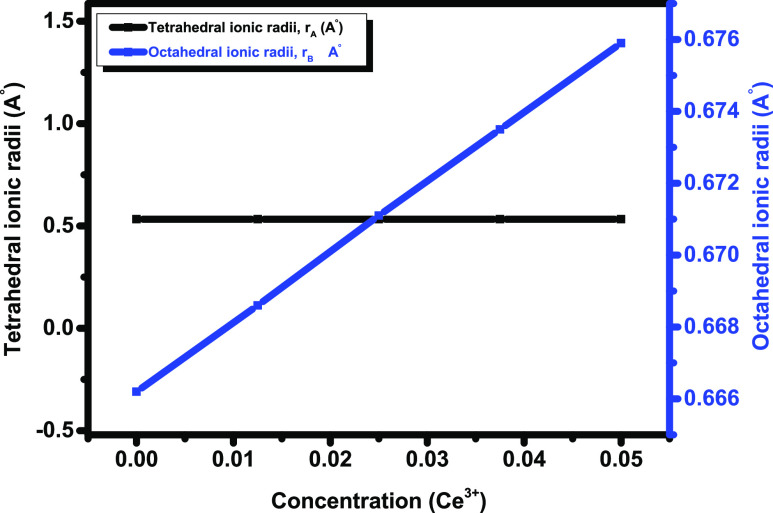
Tetrahedral and octahedral ionic radii vs cerium concentration for Ce-doped Cu–Cd ferrites.

The ionic radii *r*_A_ and *r*_B_ for each composition depend on the arrangement of cations at the A and B sublattice sites.^35−37^ Cadmium and cerium have normal and inverse spinel structures, respectively. The ionic radii were determined by arranging the compositional elements on tetrahedral and octahedral sites as follows
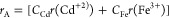
5
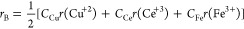
6where, *r*(Cd^2+^), *r*(Fe^3+^), *r*(Cu^2+^), and *r*(Ce^3+^) are the ionic radii of Cd^2+^, Fe^3+^, Cu^2+^, and Ce^3+^, correspondingly. The concentrations of Cu, Cd, Ce, and Fe are expressed as *C*_Cu_, *C*_Cd_, *C*_Ce_, and *C*_Fe_, respectively, in Table 4. The tetrahedral ionic (*r*_A_) radii show stable values, while the octahedral ionic radii (*r*_B_) show an increasing trend with an increase in the Ce^3+^ concentration. This is because the dopant (Ce) has a higher ionic radius than iron, as shown in Figure 3. The theoretical lattice constant (*a*_th_) was found by the following relation^38^
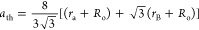
7where *R*_o_ is the ionic radius of oxygen. Figure 2 shows that *a*_th_ has higher values than the experimental lattice constant (*a*_exp_). The difference in experimental and theoretical lattice constants is due to the difference in the ionic radii of Fe^2+^ (0.67 Å) and Fe^3+^ (0.64 Å) ions.^18^ Such a difference may also be due to the presence of some external contaminations during the synthesis process. The outcomes for tolerance factor (*T*) are reported in Table 5 and were computed using the following equation
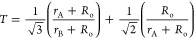
8

**Table 4:** Cation Distributions (A and B sites), Formula Weight (g/mol), and Ionic Radii (*r*_A_, *r*_B_) of Ce-Doped Cu–Cd Soft Ferrites

*X*	tetrahedral site (A)	octahedral site (B)	formula weight (g/mol)	*r*_A_ (Å)	*r*_B_ (Å)
0.0	Cd_0.5_^2+^ + Fe_0.5_^3+^	Cu_0.5_^2+^ + Ce_0_^3+^Fe_1.5_^3+^	263.664	0.5331	0.6662
0.0125	Cd_0.5_^2+^ + Fe_0.5_^3+^	Cu_0.5_^2+^ + Ce_0.0125_^3+^Fe_1.4875_^3+^	239.754	0.5331	0.6686
0.0250	Cd_0.5_^2+^ + Fe_0.5_^3+^	Cu_0.5_^2+^ + Ce_0.025_^3+^Fe_1.475_^3+^	239.360	0.5331	0.6711
0.0375	Cd_0.5_^2+^ + Fe_0.5_^3+^	Cu_0.5_^2+^ + Ce_0.0375_^3+^Fe_1.4625_^3+^	238.966	0.5331	0.6735
0.05	Cd_0.5_^2+^ + Fe_0.5_^3+^	Cu_0.5_^2+^ + Ce_0.05_^3+^Fe_1.45_^3+^	238.572	0.5331	0.6759

**Table 5:** Theoretical Lattice Constant (*a*_th_), Tolerance Factor (*T*), Oxygen Positional Parameter (*U*), and Interionic Distances (*d*_AE_, *d*_BL_, *d*_BLU_) for the Composed Nanoferrites

parameter	0.0	0.0125	0.0250	0.0375	0.050
*a*_th_ (Å)	8.3065	8.3130	8.3195	8.3260	8.3325
*T* (Å)	1.0456	1.0449	1.0442	1.0435	1.0428
*U* (Å)	0.38407	0.38401	0.38369	0.38354	0.38346
*d*_AL_ (Å)	3.1925	3.1925	3.1925	3.1925	3.1925
*d*_BL_ (Å)	2.7606	2.7631	2.7773	2.7839	2.7875
*d*_BLU_ (Å)	2.9805	2.9816	2.9885	2.9917	2.9934

The tolerance factor approaches unity, indicating that nanoferrites have defect-free spinel structures. The oxygen ion parameter (*U*) was found as follows
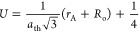
9

The ideal value of the spinel structure for the oxygen ion parameter “*U*” is about 0.375. However, after the exploration of samples, there was some fluctuation from the ideal value of the oxygen ion parameter. This fluctuation is due to the distortion of the lattice, as shown in Table 5.^39^

#### 3.1.3 Interionic Distances

The interionic distances for A and B sites are named cation–cation and anion–anion distances, respectively. The tetrahedral edge length , shared octahedral edge length (*d*_BL_ = √2 (1 – 2*u*)*a*), and unshared octahedral edge length  are the interatomic distances investigated based on ref (40). The determined values for all interionic distance parameters are presented in Table 5. The tetrahedral edge length (*d*_AL_) has the same value for all nanoferrites (*x* = 0.0–0.050) because tetrahedral ionic radii are unchanged. However, the rest of the interionic distances (*d*_BL_ and *d*_BLU_) demonstrate amplified behaviors with the addition of cerium, and this may be attributed to an increase in the ionic radii of the octahedral sites.

### 3.2 Scanning Electron Microscopy (SEM)

**Figure 4:**
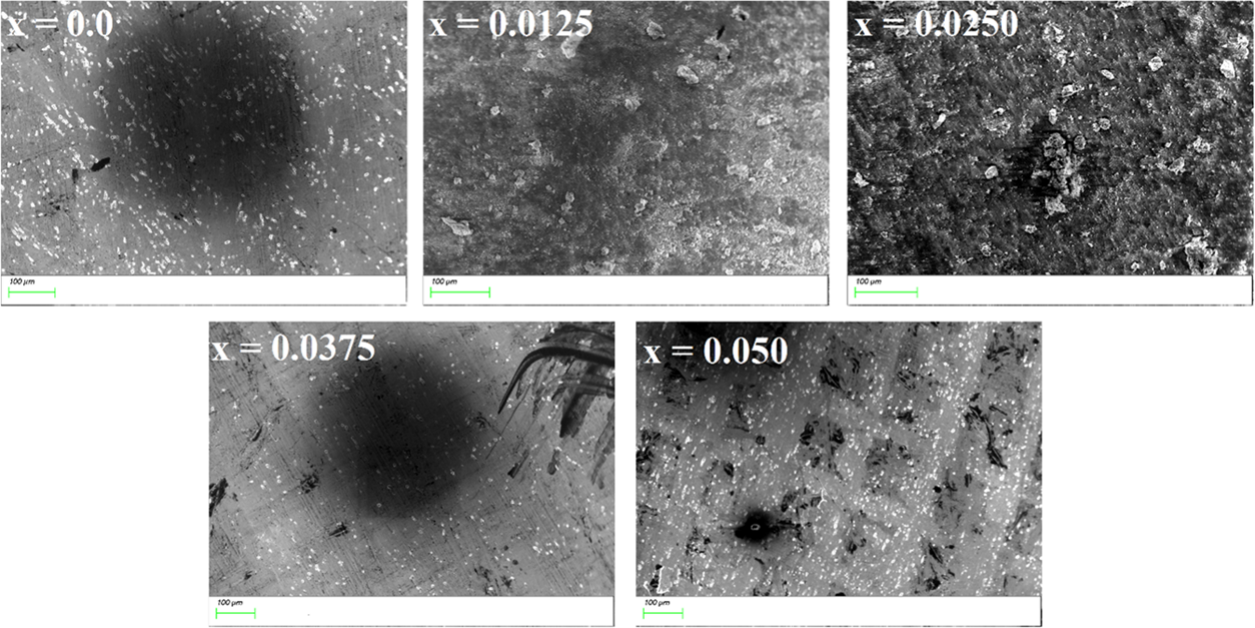
SEM images of Ce^3+^-doped Cu_0.5_Cd_0.5_Fe_2–*x*_Ce_*x*_O_4_ (*x* = 0.0, 0.0125, 0.025, 0.0375, and 0.050) soft ferrites.

Microimages obtained from SEM are displayed in Figure 4 for *x* = 0.0–0.050 nanoferrites. The images show that the nanoparticle sizes are less than 100 nm. The figure shows that samples with *x* = 0.0, 0.0375, and 0.050 appear spherical in shape and their surfaces appear to be flat.^31^ The rest of the nanoferrite samples have no apparent boundaries because of aggregation. Such a rough surface is because of the agglomeration of individual nanoferrites into aggregates.^41^ Furthermore, the size of the particles reduces with an increase in the cerium content because of lattice extensions and disorders in lattice provisions. Such a crystallization structure leads to a decrease in the crystallite size.^42,43^ Crystallites are small individual crystalline domains that make up a larger crystal structure. In ferrite materials, there are numerous crystallites that may have different orientations. This may cause differences in the crystallite and particle size.

### 3.3 Fourier Transform Infrared Spectroscopy

**Figure 5:**
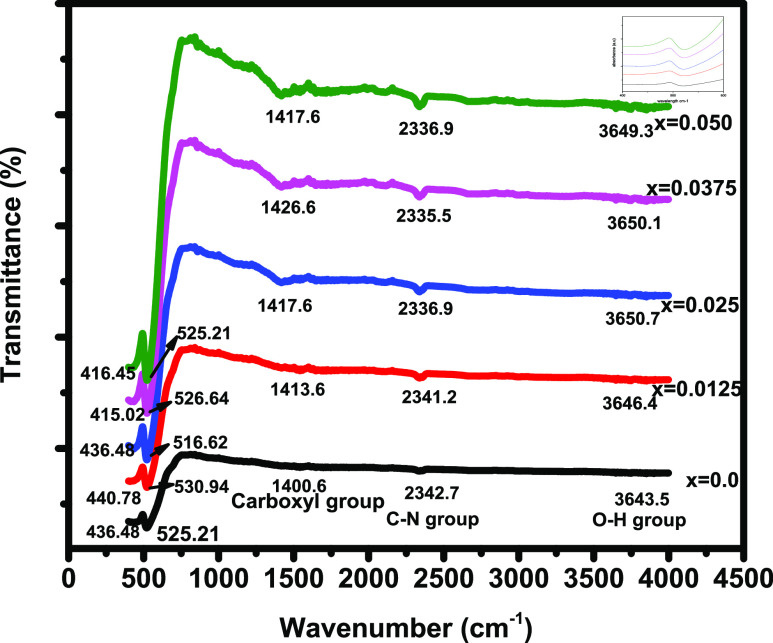
FTIR spectra of Cu_0.5_Cd_0.5_Fe_2–*x*_Ce_*x*_O_4_ (*x* = 0.0, 0.0125, 0.025, 0.0375, and 0.050) nanoferrites.

Different absorption bands at different wavelengths are studied by FTIR spectroscopy. FTIR analysis was executed to confirm the phase change in the Cu_0.5_ Cd_0.5_Ce_*x*_Fe_2_O_4_ (*x* = 0.0, 0.0125, 0.025, 0.0375, and 0.050) nanoferrites and to explain their structural properties. The FTIR spectra of Ce^3+^-doped soft ferrites in the frequency range of 400–4000 cm^–1^ are shown in Figure 5. Two ordinary absorption bands in the ferrite spectra were exhibited under 1000 cm^–1^ because of the M–O stretching vibration mode at tetrahedral and octahedral sites. The upper- (*V*_1_) and lower-frequency (*V*_2_) absorption bands^7^ lie in the ranges of 516–530 and 415–440 cm^–1^, respectively, as given in Table 6. The Fe–O bond lengths at A-sites with *R*_A_ = 1.955 Å are shorter than the bond lengths at B-sites with *R*_B_ = 2.033–2.035 Å; the trend of these values is compatible with *V*_1_ and *V*_2_, which shows nonlinearity.^44^ Furthermore, it can be observed that the specimen Cu_0.5_Cd_0.5_Fe_1.975_Ce_0.025_O_4_ shows the lowest values for both wavenumbers and intensities compared to the rest of the samples. It is due to this fact that bond lengths for tetrahedral bands (0.5331 Å) and octahedral bands (0.6711 Å) are lower than those of the other synthesized ferrites. The small peaks approximately at 1400 and 2335 cm^–1^ represent the symmetric stretching of the carboxyl group and C–N stretching of the cyanide group, respectively.^8^ The band at 3650 cm^–1^ is associated with the O–H stretching mode.^45^ The inset image in Figure 5 shows the peaks of the tetrahedral band in the FTIR spectra. The force constants (*K* = 4π^2^ν^2^*C*^2^*m*) for the tetrahedral (*K*_T_) and octahedral sites (*K*_O_) of the IR band frequency *V*_1_ and *V*_2_ were calculated as given in Table 6. The variations in bond lengths of Fe^3+^–O^2–^ in both sites are responsible for such band patterns. The entire force constants reveal variable patterns with the growth of cerium contents. An increase in the force constants is associated with the stretching of bandwidths and also the higher ionic radius of the dopant (Ce). Furthermore, the decreasing trend of the force constant is attributed to reductions in repulsive forces between ions. Similar trends were reported by Zhang et al.^46^ and Bellamy et al.^47^ It can also be observed that force constant values for Cu_0.5_Cd_0.5_Fe_1.975_Ce_0.025_O_4_ are lower than those of other nanoparticles. This behavior may be due to the fact that repulsive forces between the ions are decreased.

**Table 6:** Wavenumbers (ν_1_ and ν_2_), Intensity (*I*_1_ and *I*_2_), and Force Constant (*K*_T_ and *K*_o_) for the Composed Nanoferrites

*X*	ν_1_ (cm^–1^)	ν_2_ (cm^–1^)	*I*_1_ (%)	*I*_2_ (%)	*K*_T_ × 10^5^ (dyn/cm)	*K*_o_ × 10^5^ (dyn/cm)
0.0	525.21	436.48	0.289	0.332	1.747	2.529
0.0125	530.94	440.78	0.364	0.376	1.781	2.584
0.0250	516.62	436.48	0.248	0.286	1.747	2.447
0.0375	526.64	415.02	0.416	0.414	1.579	2.543
0.050	525.21	416.45	0.295	0.299	1.590	2.529

### 3.4 UV–Visible Spectroscopy

**Figure 6:**
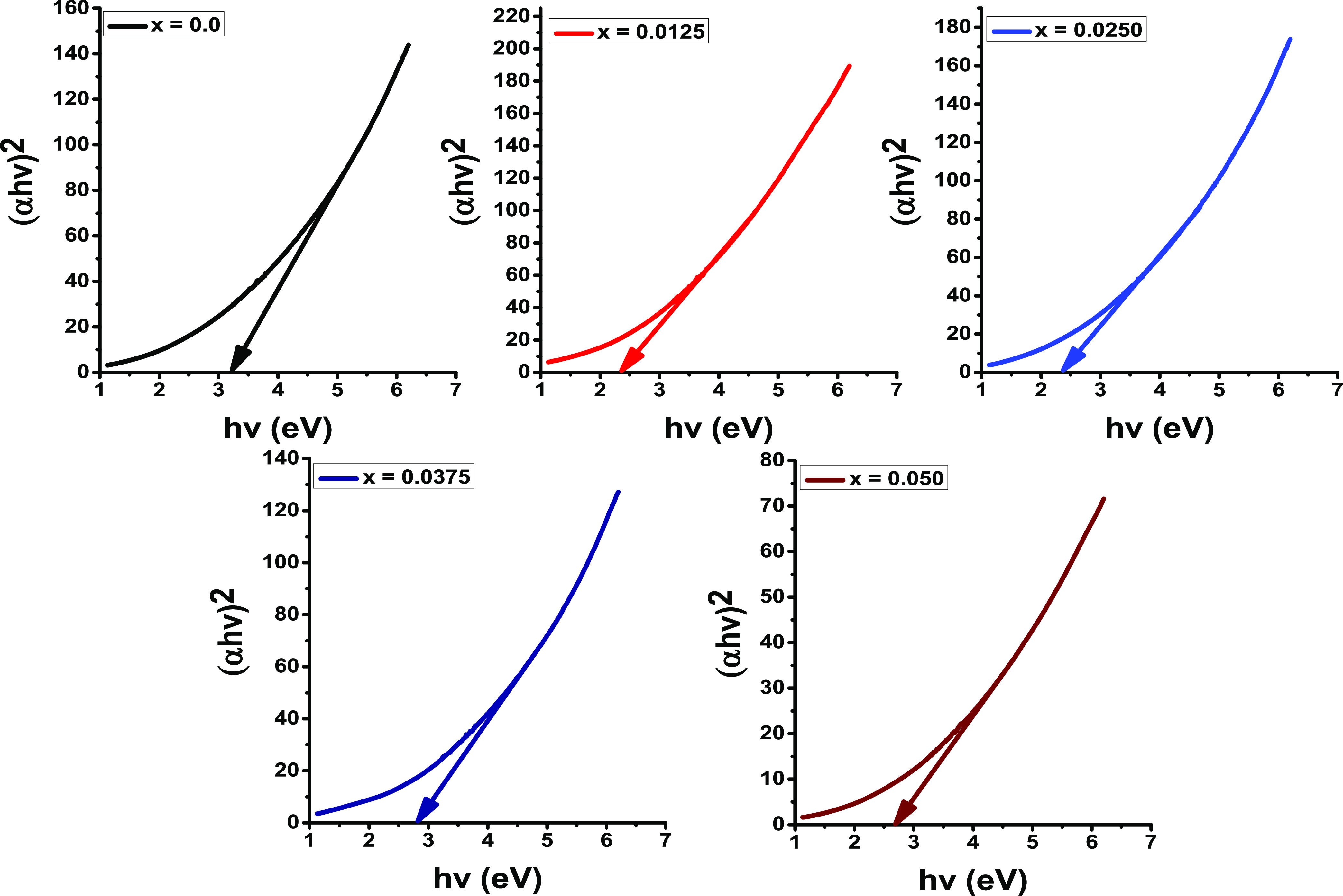
Optical bandgap energies of Ce-doped Cu–Cd ferrites.

UV–visible spectroscopy was performed to explore the optical properties of nanomaterials. Nanoparticles with reduced dimensionality and a high surface-to-volume ratio are becoming preferred candidates for optoelectronic devices. UV–visible spectroscopy is performed in the wavelength range of 200–800 nm for the absorption spectrum of Cu_0.5_Cd_0.5_Ce_*x*_Fe_2–*x*_O_4_ (*x* = 0.0, 0.0125, 0.025, 0.0375, 0.05). The bandgap energy (*E*_g_) was calculated by the Tauc’s relation^33^
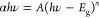
10where α and A denote the absorption coefficient and transition probability constant, respectively. *n* = 1/2 and 1 for indirect and direct band gaps, respectively.^48^ The absorption coefficient can be calculated by using the following relation
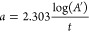
11where *A*′ is the absorbance and *t* is the width of the substance having a value of unity. *E*_g_ can be estimated by drawing a plot of (α*hv*)^2^ versus (*hv*). The tangent lying on the *x*-axis yields the value of *E*_g_, as illustrated in Figure 6. *E*_g_ lies between 3.22 and 2.68 eV for *x* = 0.0–0.050, respectively. *E*_g_ decreases with increasing Ce^3+^ content, as shown in Table 7. The bandgap energy reduces with the reduction of crystallite size; this confirms the quantum confinement of all of the composed nanoferrites. This is attributed to the polycrystalline behavior of nanomaterials composed of crystallites in the range of nanometers.^49^ The magnitudes of the optical bandgap energies confirm that the composed nanomaterials are semiconductors. Furthermore, it can be observed that Cu_0.5_Cd_0.5_Fe_1.975_Ce_0.025_O_4_ shows the lowest optical bandgap energy compared to the rest of the nanoferrites.

**Table 7:** Optical Bandgap Energy (*E*_g_), Activation Energy, and Δ*E* (Ferrimagnetic Region *E*_f_ and Paramagnetic Region *E*_p_) Values of Ce-Doped Cu–Cd Ferrites

*X*	*E*_g_ (eV)	*E*_f_ (eV)	*E*_P_ (eV)	Δ*E* = *E*_P_ – *E*_f_ (eV)
0.0	3.22	0.3943	0.3503	–0.0440
0.0125	2.34			
0.0250	2.34	0.3301	0.2940	–0.0360
0.0375	2.79	0.3725	0.4104	0.0379
0.050	2.68	0.4663	0.3876	–0.0787

### 3.5 Electrical Properties

#### 3.5.1 DC Electrical Resistivity

**Figure 7:**
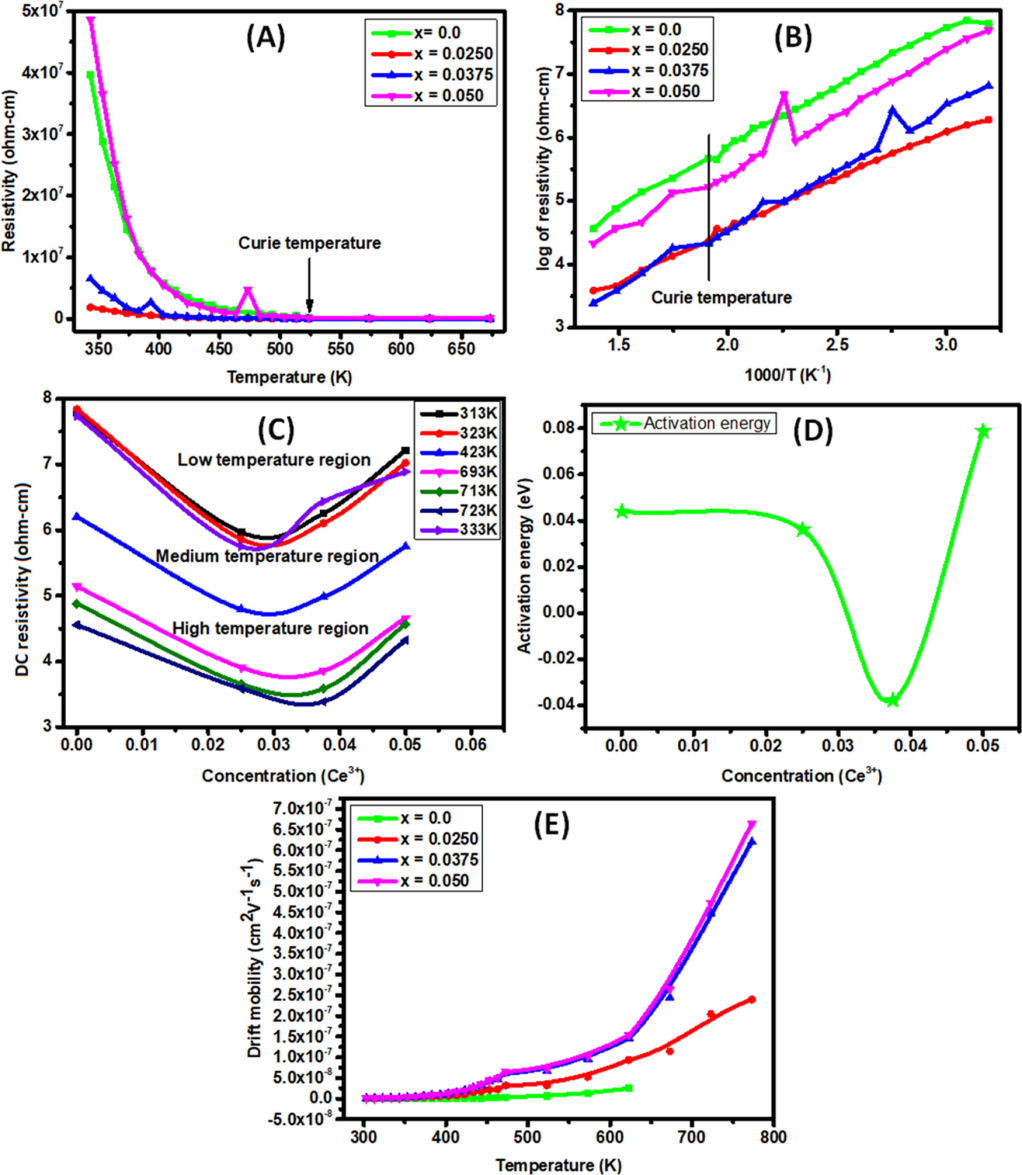
(A) Graph of DC electrical resistivity and temperature. (B) Graph of the log of DC electrical resistivity and the inverse of temperature. (C) Graph of the DC electrical resistivity and the concentration at various temperatures. (D) Graph of the activation energy and concentration. (E) Graph of the drift mobility and temperature for Ce-doped Cu–Cd ferrites (*x* = 0.0–0.050).

Electrical properties of Ce^3+^-doped copper–cadmium nanoferrites were determined using the two-probe techniques in the temperature range of 313–773 K. The equation  was used to determine the DC electrical resistivity. Resistance can be determined using the inverse slopes from current and voltage graphs. Figure 7A shows the plot of DC electrical resistivity (ρ_DC_) and temperature. The DC resistivity reduces with an increase in temperature, resulting in the soft ferrites exhibiting semiconducting behavior. The movement of thermally activated charge carriers is responsible for the loss in resistivity.^50^ The mobility of charge carriers changes with the temperature, which changes the conductivity, whereas the carrier concentration remains the same with increasing temperature. According to the hopping mechanism, the conduction of current occurs among the iron atoms because of an alteration in the charge carrier mobility with the temperature.^51^ Fe^3+^ ions exist at tetrahedral sites, so hopping does not occur at the A-site, while Fe^2+^ exists at B-sites. Hence, the conduction occurs because of the hopping of charge carriers at the octahedral site among Fe^2+^ and Fe^3+^ ions.^52^ The resistivity increases when iron is replaced by cerium because the resistivity of Ce^3+^ (75 μΩ·cm) is greater than that of Fe^3+^ (9.71 μΩ·cm).^53^ Here, Figure 7A shows that *x* = 0.0250 shows the lowest resistivity compared with the rest of the nanoferrites. Hence, the conduction process increases due to the hopping of charge carriers. The range of resistivity observed for these materials (10^6^ ohm·cm) makes them applicable in microwave device design.Figure 7 B describes the DC electrical resistivity vs the inverse of 1000/temperature. The data lines are divided into two segments split by a kink (at 523 K, known as the Curie temperature) between ferrimagnetic and paramagnetic behavior. The DC electrical resistivity decreases until the kink then becomes linear. Figure 7C shows that resistivity exhibits the opposite trend in both regions with increasing Ce^3+^ content. The conductivity is affected by the activation energy, both having an inverse relationship with each other. The activation energy can be investigated by the following formula
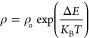
12where *K*_B_ and *T* are the Boltzmann constant and temperature, correspondingly. In Figure 7D, Δ*E* shows variable trends with increasing cerium concentration. Such a trend is due to the change in ionic distances in the crystal structure. The activation energy of the ferromagnetic region is greater than that of the paramagnetic region, as illustrated in Table 7, because in the ferromagnetic state, the domains are in the ordered form, while in the paramagnetic state, the domains are random. Hence, a large potential is essential to activate the charge carriers in ferro regions.

#### 3.5.2 Drift Mobility

The drift mobility (μ_d_) of charge carriers was estimated from the following equation
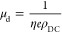
13where η is the charge carrier concentration, e is the charge on electrons, and ρ_DC_ is the DC electrical concentration. The charge carrier concentration is determined from the following equation
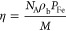
14where *N*_A_ is the Avogadro number having a value of 6.0221 × 10^23^ mol^–1^, *P*_Fe_ is the concentration of iron in composition, and *M* is the molecular weight. In Figure 7E, the increase in μ_d_ with an increase in temperature is attributed to the mobility of charge carriers due to the thermal activation mechanism.^54^ Furthermore, ρ_dc_ and μ_d_ are inversely related to each other. The intensification of the drift mobility is attributed to an increase in the hopping of charge carriers due to the increase in temperature.

### 3.6 Dielectric Properties

**Figure 8:**
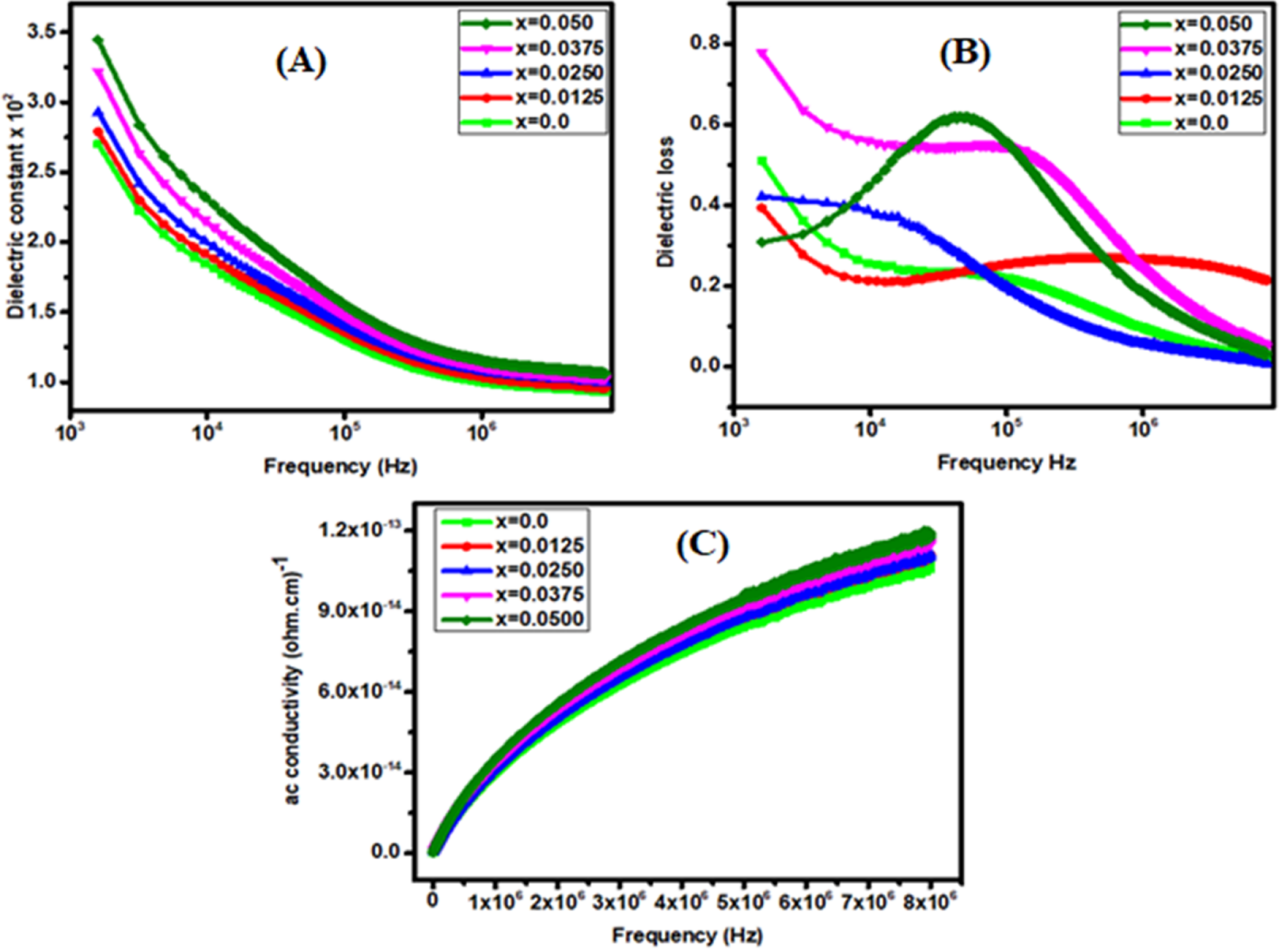
(A) Dielectric constant versus frequency. (B) Dielectric loss versus frequency. (C) AC conductivity versus frequency for Ce-doped Cu–Cd ferrites.

The dielectric properties of nanoferrites are associated with the fabrication method and distribution of cations. Lower dielectric losses and higher resistivity are favorable for the electronic properties of spinel ferrites. Therefore, high-quality nanocomposites are synthesized by the application of an economical procedure. In Figure 8A, the dielectric constant gradually decreases with the frequency due to the hopping of electrons between Fe^2+^ and Fe^3+^ ions, which caused the polarization.^55^ For cerium-doped soft ferrites, the iron ions are substituted by cerium. The decrease in Fe^3+^ ions at A-sites may be caused by the decrease in the dielectric constant. The enhancement in Ce^3+^ ions causes a reduction in polarization and leads to a decrease in the dielectric constant. Furthermore, the dielectric constant also decreases with an increase in the frequency. In Ce-doped Cu–Cd ferrites, the dielectric constant decreases rapidly from 10 kHz to 1 MHz and then attains a constant value for the rest of the frequency range, as shown in Figure 8A. Soft ferrites were supposed to consist of dual layers. The primary layer is composed of well-conducting grains, followed by a combination of highly resistive grain boundaries. This causes charge localization through the applied field, which causes interfacial polarization. This applied field is not followed by the movement of ions among iron ions, which decreases the interfacial polarization. Such outcomes decrease the dielectric constant. Melagiriyappa and Ajmal et al.^56,57^ reported similar outcomes. Figure 8B shows that the dielectric loss “tan δ” decreases with an increase in the cerium content. This may be due to an increase in resistance with the increasing cerium content. Soft ferrites are combinations of fine conducting grains that are divided by poorly conducting grain boundaries. In lower-frequency areas, these grain boundaries are more efficient. Therefore, for the hopping mechanism, a large amount of energy is required. In low-frequency regions, the energy loss increases. Hence, the dielectric loss is higher in the low-frequency region. Fine conducting grains are more efficient in the higher-frequency regions. For the hopping process, low energy is needed to shift the charge carriers, so the dielectric loss is lower at higher frequencies.^31^ Furthermore, a peak appears in the frequency regions between 10^4^ and 10^5^ Hz. This peak becomes stronger with the increase in cerium concentration. The appearance of the peak might be because of the fact that energy loss increases due to hopping. In addition, it can be noticed that for sample Cu_0.5_Cd_0.5_Fe_1.975_Ce_0.025_O_4_ (*x* = 0.025), the dielectric losses decrease linearly with an increase in frequency. This sample demonstrated the lowest dielectric losses sharply after 6 × 10^4^ Hz frequency. This behavior leads to a decrease in the energy loss. Hence, this cerium-doped specimen has become the most valuable candidate in the case of the lowest energy losses as compared to others. This behavior is confirmed in Figure 7A, as the lowest resistivity is shown by the specimen. Figure 8C shows the behavior of AC conductivity against the frequency for *x* = 0.0–0.050 nanoferrites. Therefore, the AC conductivity increased with increasing frequency. Such a nature of AC conductivity is attributed to empty spaces and interactions between cerium and iron atoms.^58^

**Figure 9:**
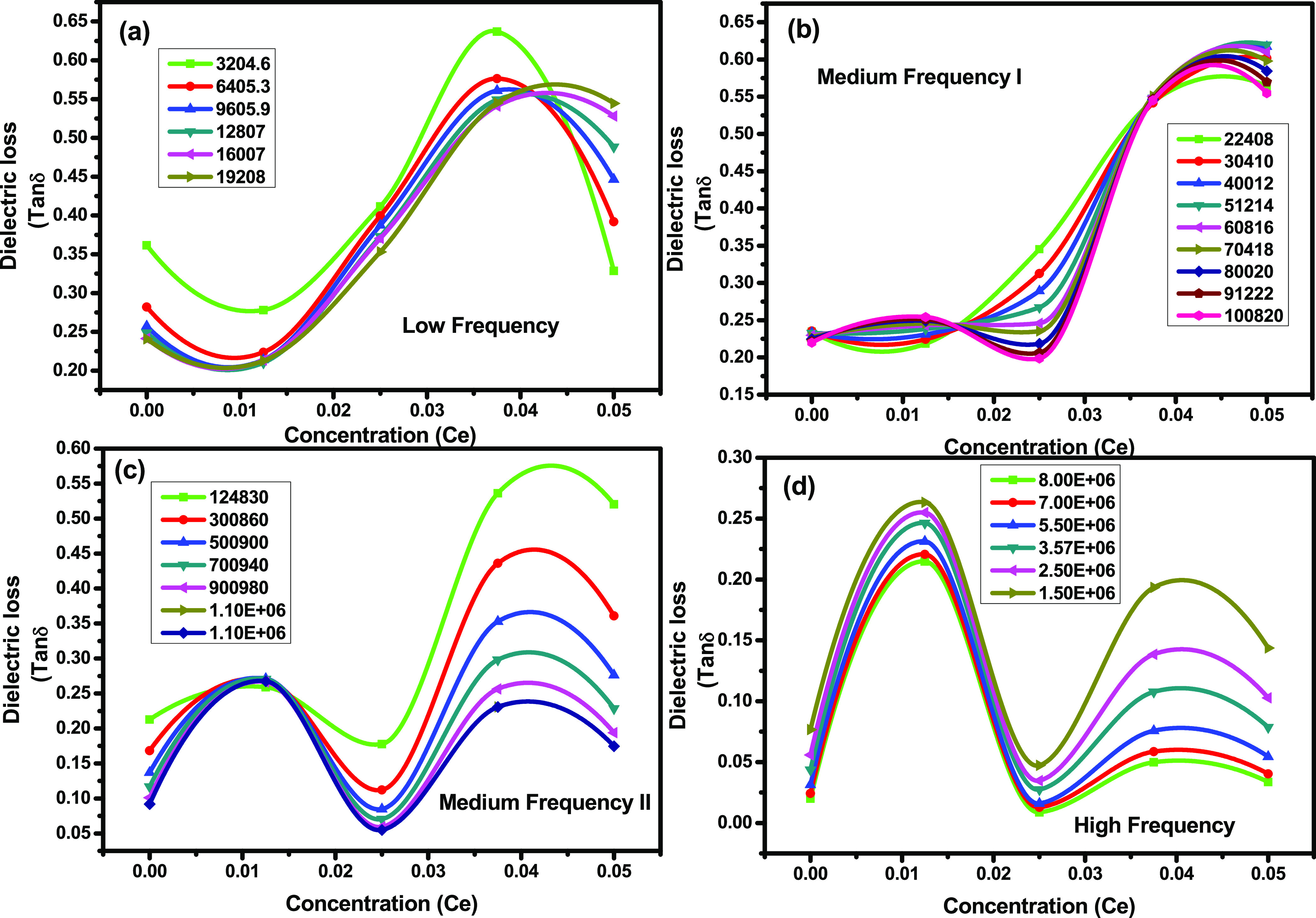
(a–d) Dielectric loss versus concentration (Ce^3+^) in (a) low-frequency regions, (b) medium-frequency region-I, (c) medium-frequency region-II, and (d) high-frequency regions for Ce-doped Cu–Cd ferrites.

**Figure 10:**
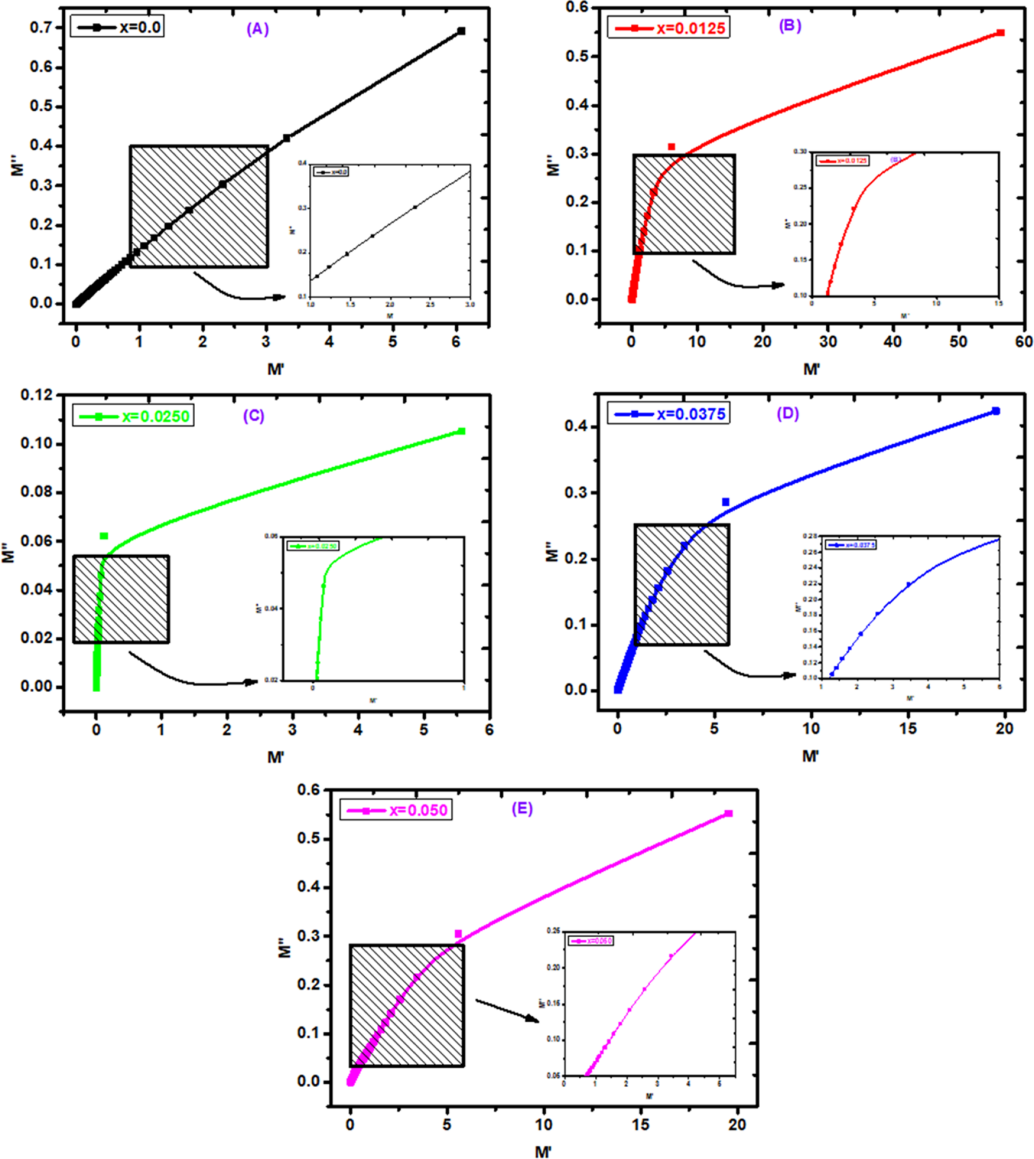
(A–E) Cole–Cole graph for Ce-doped Cu–Cd ferrites.

Figure 9a–d shows the relation between dielectric loss (tan δ) and cerium concentration in various frequency regions. Figure 9a–d shows that *x* = 0.0 shows the lowest tan δ for all frequency regions. The dielectric loss for *x* = 0.0125 increases with increasing frequency and shows the maximum values in high-frequency regions. For *x* = 0.0250, the dielectric losses are reduced with the increase in frequency. This is because a lower energy is desirable to shift the charge carriers during hopping. Hence, with an increase in the frequency, the dielectric losses decrease.^31^ This behavior of Cu_0.5_Cd_0.5_Fe_1.975_Ce_0.025_O_4_ (*x* = 0.0250) is in good agreement with Figure 7A, which describes the behavior of the DC electrical resistivity. For *x* = 0.0375, the dielectric losses again show a reducing trend with increasing frequency. For *x* = 0.050, the tan δ increases with an increase in frequency in low-frequency and medium-frequency region-I. However, in medium-frequency region-II and in higher-frequency regions, it again decreases. Figure 9a–d shows variable trends with increasing cerium concentration. Figure 9 shows that tan δ decreases for *x* = 0.0, 0.0125, and 0.0375 with an increase in the cerium content. This is attributed to the fact that the interaction between Fe–Fe ions reduces.^59^ However, (Cu_0.5_Cd_0.5_Fe_1.975_Ce_0.025_O_4_) *x* = 0.0250 also shows a decreasing trend, but the line is linear. The behavior shown by *x* = 0.050 is totally opposite to those of the rest of the samples and has an increasing nature. Murthy and Sobhandri explained this behavior on the basis of polarization. They concluded that exchanges of electrons among Fe^2+^–Fe^3+^ ions are associated with displacements to investigate the polarization in ferrites.^60^ This result shown by the Cu_0.5_Cd_0.5_Fe_1.975_Ce_0.025_O_4_ is quite in agreement with the outcomes of DC electrical resistivity. Figure 10A–E shows the Cole–Cole graph for Ce-doped Cu–Cd ferrites. The Cole–Cole graph gives information about the conductive parts of grains to resistive interfaces. In Figure 10, a prominent bend is shown by the synthesized ferrites (B–E), confirming that the resistive interfaces are stronger than the conduction effects of grains. The lack of semicircles for all ferrites indicates that the conduction effect of grains is more dominant than the resistive effect of the interfaces. The appearance of such a modulus spectrum indicates that the conduction mechanism exhibits a non-Debye relaxation.^61^

## Conclusions

In the current research work, the chemical compositions Cu_0.5_Cd_0.5_Fe_2–*x*_Ce_*x*_O_4_ (*x* = 0.0, 0.0125, 0.025, 0.0375, 0.050) were prepared by a hydrothermal route. The substitution of cerium reduced the crystallite size. The lattice constant showed an increasing trend due to variations in the ionic radii of cerium and iron. Various other parameters, including densities, ionic radii, tolerance factor, positional parameter for oxygen, bond lengths, etc., were also determined by XRD. SEM images were obtained to investigate the surface shape of nanoparticles. Tetrahedral and octahedral bands were evaluated in the ranges of 516–530 and 415–440 cm^–1^, respectively. The bandgap energy increased from 2.68 to 3.44 eV, confirming the semiconducting nature of the soft ferrites. The DC electrical resistivity exhibited a reducing trend with an increase in temperature. Further, the dielectric parameters also showed decreasing trends due to polarization. Cu_0.5_Cd_0.5_Fe_1.975_Ce_0.025_O_4_ showed the least optical bandgap energy, DC electrical resistivity, and dielectric losses. On the basis of these electrical and dielectric parameters, it can be recommended that the composed materials are highly applicable in the fabrication of microwave devices.
